# Supplementary material for: Modeling glioblastoma heterogeneity as a dynamic network of cell states
Source: Mol Syst Biol. 2021 Sep 16;17(9):e10105. doi: 10.15252/msb.202010105 (PMC8444284; doi:10.15252/msb.202010105)
Supplement: Supplementary file 5 — Source Data for Figure 3 [file MSB-17-e10105-s001.zip › Figure3A_sourcedata/GSEA_3065/nosigdb_state6.GseaPreranked.1622718443993/FELDMAN_2020_G1.html]

Details for gene set FELDMAN\_2020\_G1[GSEA]

|  || Dataset | state6 |
| Phenotype | NoPhenotypeAvailable |
| Upregulated in class | na\_neg |
| GeneSet | FELDMAN\_2020\_G1 |
| Enrichment Score (ES) | -0.61137813 |
| Normalized Enrichment Score (NES) | -2.59292 |
| Nominal p-value | 0.0 |
| FDR q-value | 0.0 |
| FWER p-Value | 0.0 |
Table: GSEA Results Summary

  

Fig 1: Enrichment plot: FELDMAN\_2020\_G1      
 Profile of the Running ES Score & Positions of GeneSet Members on the Rank Ordered List

  

| PROBE | GENE SYMBOL | GENE\_TITLE | RANK IN GENE LIST | RANK METRIC SCORE | RUNNING ES | CORE ENRICHMENT || 1 | PTMA |  |  | 94 | 0.216 | 0.0040 | No |
| 2 | S100B |  |  | 391 | 0.137 | -0.0209 | No |
| 3 | HMGN2 |  |  | 679 | 0.108 | -0.0467 | No |
| 4 | HIST1H1C |  |  | 768 | 0.101 | -0.0499 | No |
| 5 | H2AFV |  |  | 782 | 0.100 | -0.0446 | No |
| 6 | NUCKS1 |  |  | 1066 | 0.082 | -0.0717 | No |
| 7 | TMEM106C |  |  | 1286 | 0.072 | -0.0921 | No |
| 8 | CENPH |  |  | 1340 | 0.070 | -0.0935 | No |
| 9 | PSIP1 |  |  | 1372 | 0.068 | -0.0924 | No |
| 10 | DEK |  |  | 1535 | 0.063 | -0.1068 | No |
| 11 | STMN1 |  |  | 1649 | 0.058 | -0.1158 | No |
| 12 | USP1 |  |  | 1712 | 0.056 | -0.1192 | No |
| 13 | TUBA1B |  |  | 2087 | 0.045 | -0.1593 | No |
| 14 | CENPA |  |  | 2199 | 0.042 | -0.1693 | No |
| 15 | C12orf75 |  |  | 2218 | 0.041 | -0.1686 | No |
| 16 | PTTG1 |  |  | 2304 | 0.038 | -0.1758 | No |
| 17 | CMC2 |  |  | 2424 | 0.035 | -0.1871 | No |
| 18 | RPA3 |  |  | 2563 | 0.032 | -0.2009 | No |
| 19 | ANP32E |  |  | 2570 | 0.032 | -0.1994 | No |
| 20 | LSM3 |  |  | 2673 | 0.029 | -0.2092 | No |
| 21 | MZT1 |  |  | 2946 | 0.023 | -0.2391 | No |
| 22 | TMPO |  |  | 2978 | 0.022 | -0.2411 | No |
| 23 | CKS1B |  |  | 2985 | 0.022 | -0.2403 | No |
| 24 | PLK1 |  |  | 3116 | 0.020 | -0.2540 | No |
| 25 | DTYMK |  |  | 3271 | 0.016 | -0.2707 | No |
| 26 | HMGB3 |  |  | 3377 | 0.014 | -0.2819 | No |
| 27 | DUT |  |  | 3596 | 0.009 | -0.3064 | No |
| 28 | HIST1H1E |  |  | 3635 | 0.008 | -0.3103 | No |
| 29 | PHF19 |  |  | 3654 | 0.008 | -0.3118 | No |
| 30 | CENPW |  |  | 3936 | 0.002 | -0.3442 | No |
| 31 | CDC20 |  |  | 3996 | 0.001 | -0.3510 | No |
| 32 | SNRPB |  |  | 4387 | -0.007 | -0.3956 | No |
| 33 | MCM7 |  |  | 4435 | -0.008 | -0.4005 | No |
| 34 | SNRPD1 |  |  | 4477 | -0.008 | -0.4047 | No |
| 35 | CCNB2 |  |  | 4736 | -0.013 | -0.4336 | No |
| 36 | DDX39A |  |  | 4809 | -0.014 | -0.4409 | No |
| 37 | ANP32B |  |  | 4891 | -0.016 | -0.4492 | No |
| 38 | CALM2 |  |  | 5062 | -0.020 | -0.4675 | No |
| 39 | CENPM |  |  | 5089 | -0.020 | -0.4691 | No |
| 40 | GTSE1 |  |  | 5195 | -0.022 | -0.4797 | No |
| 41 | HIST1H1B |  |  | 5374 | -0.026 | -0.4986 | No |
| 42 | MIAT |  |  | 5393 | -0.026 | -0.4988 | No |
| 43 | SCRG1 |  |  | 5450 | -0.027 | -0.5034 | No |
| 44 | H2AFX |  |  | 5451 | -0.027 | -0.5016 | No |
| 45 | LMNB1 |  |  | 5545 | -0.029 | -0.5103 | No |
| 46 | BUB3 |  |  | 5807 | -0.034 | -0.5381 | No |
| 47 | KNSTRN |  |  | 5875 | -0.036 | -0.5434 | No |
| 48 | PDLIM7 |  |  | 6033 | -0.039 | -0.5589 | No |
| 49 | NUDT1 |  |  | 6071 | -0.040 | -0.5605 | No |
| 50 | H2AFZ |  |  | 6079 | -0.040 | -0.5586 | No |
| 51 | CYCS |  |  | 6090 | -0.040 | -0.5570 | No |
| 52 | CDCA2 |  |  | 6175 | -0.042 | -0.5638 | No |
| 53 | CENPN |  |  | 6194 | -0.042 | -0.5630 | No |
| 54 | AURKB |  |  | 6215 | -0.043 | -0.5623 | No |
| 55 | DNAJC9 |  |  | 6230 | -0.043 | -0.5610 | No |
| 56 | CDKN3 |  |  | 6291 | -0.044 | -0.5649 | No |
| 57 | RANBP1 |  |  | 6309 | -0.045 | -0.5638 | No |
| 58 | GINS2 |  |  | 6461 | -0.049 | -0.5779 | No |
| 59 | SRSF7 |  |  | 6593 | -0.053 | -0.5894 | No |
| 60 | PTGES3 |  |  | 6602 | -0.053 | -0.5867 | No |
| 61 | VRK1 |  |  | 6626 | -0.053 | -0.5857 | No |
| 62 | TROAP |  |  | 6707 | -0.056 | -0.5911 | No |
| 63 | MKI67 |  |  | 6750 | -0.057 | -0.5921 | No |
| 64 | PRC1 |  |  | 6918 | -0.062 | -0.6071 | Yes |
| 65 | HNRNPDL |  |  | 6956 | -0.063 | -0.6070 | Yes |
| 66 | RAD21 |  |  | 6984 | -0.064 | -0.6057 | Yes |
| 67 | IGFBP5 |  |  | 7011 | -0.065 | -0.6043 | Yes |
| 68 | BIRC5 |  |  | 7024 | -0.066 | -0.6012 | Yes |
| 69 | SIVA1 |  |  | 7054 | -0.066 | -0.6000 | Yes |
| 70 | CKAP2L |  |  | 7065 | -0.067 | -0.5966 | Yes |
| 71 | KIF22 |  |  | 7098 | -0.068 | -0.5956 | Yes |
| 72 | MAP3K7CL |  |  | 7100 | -0.068 | -0.5911 | Yes |
| 73 | PLP1 |  |  | 7138 | -0.069 | -0.5906 | Yes |
| 74 | HMGB1 |  |  | 7186 | -0.071 | -0.5912 | Yes |
| 75 | FEN1 |  |  | 7218 | -0.071 | -0.5899 | Yes |
| 76 | RAN |  |  | 7221 | -0.072 | -0.5853 | Yes |
| 77 | ZWINT |  |  | 7370 | -0.077 | -0.5971 | Yes |
| 78 | TOP2A |  |  | 7430 | -0.079 | -0.5985 | Yes |
| 79 | RRM1 |  |  | 7491 | -0.081 | -0.5999 | Yes |
| 80 | PRCP |  |  | 7495 | -0.081 | -0.5947 | Yes |
| 81 | TK1 |  |  | 7499 | -0.081 | -0.5895 | Yes |
| 82 | SPC25 |  |  | 7522 | -0.082 | -0.5864 | Yes |
| 83 | HAT1 |  |  | 7538 | -0.083 | -0.5824 | Yes |
| 84 | CCDC34 |  |  | 7609 | -0.087 | -0.5846 | Yes |
| 85 | CDCA3 |  |  | 7631 | -0.087 | -0.5810 | Yes |
| 86 | DEPDC1 |  |  | 7637 | -0.087 | -0.5756 | Yes |
| 87 | DBF4 |  |  | 7639 | -0.088 | -0.5697 | Yes |
| 88 | HIST1H4C |  |  | 7641 | -0.088 | -0.5639 | Yes |
| 89 | GMNN |  |  | 7651 | -0.088 | -0.5588 | Yes |
| 90 | LSM5 |  |  | 7653 | -0.088 | -0.5529 | Yes |
| 91 | EXOSC8 |  |  | 7671 | -0.089 | -0.5487 | Yes |
| 92 | AURKA |  |  | 7700 | -0.091 | -0.5457 | Yes |
| 93 | NDC80 |  |  | 7734 | -0.093 | -0.5432 | Yes |
| 94 | ESCO2 |  |  | 7778 | -0.094 | -0.5417 | Yes |
| 95 | UBE2S |  |  | 7818 | -0.097 | -0.5396 | Yes |
| 96 | PBK |  |  | 7905 | -0.101 | -0.5426 | Yes |
| 97 | NUF2 |  |  | 7936 | -0.103 | -0.5390 | Yes |
| 98 | HNRNPA2B1 |  |  | 7941 | -0.103 | -0.5324 | Yes |
| 99 | CCNB1 |  |  | 7958 | -0.104 | -0.5271 | Yes |
| 100 | CEP55 |  |  | 7982 | -0.106 | -0.5225 | Yes |
| 101 | RRM2 |  |  | 8001 | -0.107 | -0.5172 | Yes |
| 102 | KPNA2 |  |  | 8045 | -0.110 | -0.5147 | Yes |
| 103 | PCNA |  |  | 8061 | -0.111 | -0.5088 | Yes |
| 104 | CKS2 |  |  | 8077 | -0.112 | -0.5028 | Yes |
| 105 | DLG1 |  |  | 8081 | -0.112 | -0.4955 | Yes |
| 106 | TPST2 |  |  | 8124 | -0.116 | -0.4924 | Yes |
| 107 | HSP90AA1 |  |  | 8153 | -0.117 | -0.4876 | Yes |
| 108 | CYR61 |  |  | 8178 | -0.120 | -0.4822 | Yes |
| 109 | ANLN |  |  | 8181 | -0.120 | -0.4742 | Yes |
| 110 | MIS18A |  |  | 8221 | -0.124 | -0.4702 | Yes |
| 111 | CCNA2 |  |  | 8227 | -0.124 | -0.4623 | Yes |
| 112 | TYMS |  |  | 8287 | -0.131 | -0.4602 | Yes |
| 113 | KIF4A |  |  | 8300 | -0.132 | -0.4525 | Yes |
| 114 | FBXO5 |  |  | 8310 | -0.133 | -0.4444 | Yes |
| 115 | CKAP2 |  |  | 8330 | -0.136 | -0.4373 | Yes |
| 116 | CDK1 |  |  | 8351 | -0.139 | -0.4301 | Yes |
| 117 | ATAD2 |  |  | 8359 | -0.141 | -0.4213 | Yes |
| 118 | TPX2 |  |  | 8370 | -0.143 | -0.4126 | Yes |
| 119 | HMGB2 |  |  | 8388 | -0.145 | -0.4047 | Yes |
| 120 | NCAPG |  |  | 8390 | -0.145 | -0.3948 | Yes |
| 121 | CENPU |  |  | 8403 | -0.147 | -0.3862 | Yes |
| 122 | AHNAK2 |  |  | 8411 | -0.148 | -0.3768 | Yes |
| 123 | MAD2L1 |  |  | 8427 | -0.150 | -0.3683 | Yes |
| 124 | CLSPN |  |  | 8440 | -0.151 | -0.3594 | Yes |
| 125 | DLGAP5 |  |  | 8447 | -0.152 | -0.3497 | Yes |
| 126 | KIF23 |  |  | 8465 | -0.154 | -0.3410 | Yes |
| 127 | ORC6 |  |  | 8482 | -0.157 | -0.3321 | Yes |
| 128 | UBE2T |  |  | 8489 | -0.159 | -0.3219 | Yes |
| 129 | NUSAP1 |  |  | 8523 | -0.167 | -0.3143 | Yes |
| 130 | TUBB4B |  |  | 8554 | -0.173 | -0.3059 | Yes |
| 131 | KIF20B |  |  | 8576 | -0.180 | -0.2960 | Yes |
| 132 | ILF2 |  |  | 8579 | -0.181 | -0.2838 | Yes |
| 133 | DNMT1 |  |  | 8582 | -0.184 | -0.2714 | Yes |
| 134 | DHFR |  |  | 8590 | -0.186 | -0.2595 | Yes |
| 135 | IGFBP3 |  |  | 8615 | -0.197 | -0.2488 | Yes |
| 136 | ARL6IP1 |  |  | 8628 | -0.203 | -0.2362 | Yes |
| 137 | ASPM |  |  | 8657 | -0.215 | -0.2247 | Yes |
| 138 | SMC2 |  |  | 8658 | -0.216 | -0.2099 | Yes |
| 139 | CENPK |  |  | 8666 | -0.220 | -0.1957 | Yes |
| 140 | GGH |  |  | 8702 | -0.239 | -0.1833 | Yes |
| 141 | CENPF |  |  | 8703 | -0.240 | -0.1668 | Yes |
| 142 | CTGF |  |  | 8715 | -0.254 | -0.1507 | Yes |
| 143 | CENPE |  |  | 8734 | -0.270 | -0.1342 | Yes |
| 144 | UBE2C |  |  | 8748 | -0.286 | -0.1162 | Yes |
| 145 | SMC4 |  |  | 8764 | -0.307 | -0.0969 | Yes |
| 146 | PTN |  |  | 8789 | -0.426 | -0.0704 | Yes |
| 147 | SCG2 |  |  | 8793 | -0.455 | -0.0396 | Yes |
| 148 | MALAT1 |  |  | 8796 | -0.581 | -0.0000 | Yes |
Table: GSEA details [plain text format]

  

Fig 2: FELDMAN\_2020\_G1: Random ES distribution      
 Gene set null distribution of ES for **FELDMAN\_2020\_G1**
